# Leaf Removal Applied to a Sprawling Canopy to Regulate Fruit Ripening in Cabernet Sauvignon

**DOI:** 10.3390/plants10051017

**Published:** 2021-05-19

**Authors:** Patrick O’Brien, Cassandra Collins, Roberta De Bei

**Affiliations:** 1 Waite Research Institute, School of Agriculture, Food and Wine, The University of Adelaide, PMB 1, Glen Osmond, SA 5064, Australia; patrick.obrien@adelaide.edu.au (P.O.); cassandra.collins@adelaide.edu.au (C.C.); 2 ARC Industrial Transformation Training Centre for Innovative Wine Production, Waite Research Institute, PMB 1, Glen Osmond, SA 5064, Australia

**Keywords:** grapevine, delayed ripening, shoot trimming, canopy management, leaf area

## Abstract

Under the effects of climate change, it is becoming increasingly common to observe excessively fast grape sugar accumulation while phenolic and flavour development are lagging behind. The aim of this research was to quantify the impacts of three different leaf removal techniques on the canopy architecture and ripening of Cabernet Sauvignon trained in a sprawl trellis system. Treatments were performed at veraison (~14 °Brix) and included (i) control; (ii) leaf plucking in the bunch zone; (iii) leaf plucking the top two-thirds of shoots, apical to the bunches; and (iv) shoot trimming. On the date of harvest, no significant difference in total soluble solids was observed between treatments. Other results including the effect of the treatments on fruit acidity, anthocyanins, phenolics, and tannins were somewhat inconclusive. While various other studies have shown the potential of leaf removal to achieve slower grape sugar accumulation without affecting the concentration of anthocyanins, phenolics, and tannins, the results of this study do not indicate a decrease in the rate of grape sugar accumulation as a result of the investigated defoliation techniques. Given the cost of implementing these treatments, the results of this study do not support the use of these methods for the purpose of delaying fruit ripening in a hot Australian climate.

## 1. Introduction

The decision of when to harvest is an important consideration in the winemaking process and is based on a multitude of factors including sugar and acidity levels, phenolic maturity, and aromatic and flavour ripeness [1]. Today, viticulturists in many wine-producing regions are facing new environmental challenges in this regard as a consequence of increasing global temperature, CO_2_ concentration, and solar radiation [2,3,4]. Harvest dates are advancing and becoming more compressed, meaning grapes are often picked at higher temperatures when compared to historic dates for their cultivars, with undesirable chemical profiles due to increased sugar concentration and potential wine alcohol level, low acidity, and undeveloped or unbalanced flavour components [5]. This trend towards overly fast ripening is associated with a decoupling between sugar accumulation and phenolic maturity, and it is becoming increasingly common to observe excessively fast sugar accumulation while anthocyanin and tannin development are lagging behind [6,7].

Because of the problems facing winemakers in this scenario, new vineyard management strategies are being explored to mitigate these negative impacts, with the aim of delaying sugar ripening, allowing for the accumulation of greater levels of tannins, anthocyanins, and other flavour components before picking [8,9,10,11]. Leaf removal is a useful management strategy to this end, as along with being a technique to balance the ratio between fruit load and vegetation, it enables the manipulation of canopy microclimate, allowing for some important grape quality improvements [12,13]. The rate of grape sugar accumulation is dependent on the ratio of leaf area to yield (LA/Y), and sugar accumulation can be delayed by reducing leaf area, effectively manipulating the source/sink ratio of the vine [14,15]. This strategy is also simple and suitable to mechanisation, particularly if the foliage being removed is spatially separated from the bunches [16].

The traditional method of defoliation involves removing leaves from the basal portion of the shoots, around the fruit zone, reducing leaf photosynthetic area, and increasing sunlight exposure and air circulation, with the aim of improving canopy microclimate and grape and wine composition [17]. The impact of this method on ripening has had varying reports, seeming to differ with the timing and severity of application, as well as cultivar, initial fruit microclimate, and choice of leaves removed [16,17]. With this method, it is important to be conscious of the amount of foliage being removed, especially if there is a risk of major heat events, as over-exposed fruit may be subject to sunburn damage [18], and extreme temperatures may inhibit anthocyanin accumulation [19]. There is also the risk of crop damage if using machinery for the removal process, as the leaves being removed are in close proximity to the fruit.

Defoliation of shoots apical to the bunches is a relatively new method of leaf removal that has shown promising results in some regions for delaying grape sugar accumulation while maintaining the same rate of production of phenolic compounds including anthocyanins and tannins [7]. This technique is typically performed post-veraison (12–15 °Brix) and involves the creation of a leafless window of approximately 50 cm by removing 30–35% of the leaves from the upper two-thirds of each shoot [1,20]. Several leaves are retained at the apex of each shoot, as well as a few leaves immediately above the fruit zone, reducing photosynthetic activity without having a significant impact on bunch sunlight exposure. Results from several studies have suggested that this could be an effective management strategy for slowing sugar accumulation while having a minimal effect on other grape components, and without affecting vine carbohydrate reserve storage [1,16]. Additionally, the location of the leaves being removed makes this strategy particularly suitable for mechanisation.

Shoot trimming is a common industry practice used to balance vine vigour and maintain canopy architecture, as well as having the added benefit of improving canopy microclimate and spray penetration. The effect of trimming on ripening depends not only on the intensity of the trimming, but also on its timing, as trimming can initiate a competitive growth of lateral shoots if performed early enough in the growing season. This method shows promise for reducing grape sugar accumulation rate, driven both by a significant reduction in LA/Y as well as carbon competition between developing laterals and the accumulation of grape sugar [16]. Issues can arise, however, if the trimming is performed too late in the season, where there is insufficient time for the loss of leaf area to be compensated with new lateral growth [21].

This study assessed the impacts of the three aforementioned leaf removal techniques on the canopy architecture and ripening of Cabernet Sauvignon in a hot Australian climate. A recent study has yielded results indicating that apical leaf removal may not be consistently effective in delaying sugar ripening in other cultivars in the same climate [22]. Since little is known about the impact of these leaf removal methods on berry sensory characteristics, berry sensory assessment was included as part of the trial. Understanding the impact of different leaf removal techniques on grape ripening could provide vineyard managers with a canopy management strategy suitable for regulating sugar accumulation, phenolic maturity and flavour ripeness, thereby helping to mitigate the negative effects of climate change and maintain grape quality for the production of premium wines.

## 2. Results

### 2.1. Climatic Conditions

**Figure 1:**
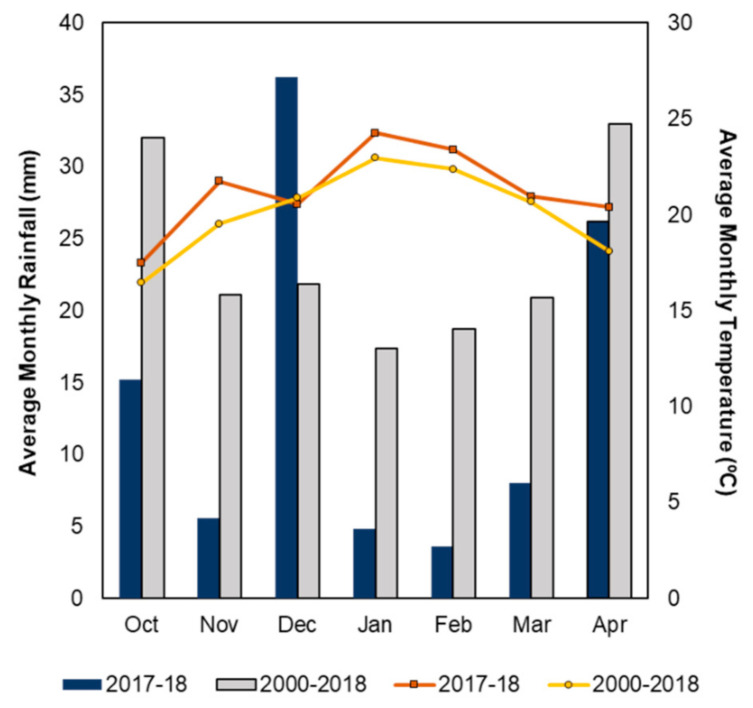
Average monthly temperature and rainfall calculated for the 2017–2018 growing season October to April and the long-term average (2000–2018). Climate data were sourced from the nearest Australian Bureau of Meteorology (http://www.bom.gov.au/, accessed on 10 June 2018) weather station located in Noarlunga (station number 23885).

Mean average temperature and rainfall for the growing season (October to April) were calculated for the long-term average (LTA; 2000–2018) and for the growing season of interest (2017–2018). The Growing Degree Days (GDD) were calculated after Gladstones [23] with base 10 and 19 °C cut offs. The 2017–2018 season was warmer than the LTA, with 1872 GDD compared to the average of 1818. The total rainfall during the 2017–2018 season was calculated as 100 mm which is considerably lower than the LTA of 165 mm. December was, however, a notably wet month, with 36 mm of rain and an average monthly temperature of 20.5 °C. A summary of the meteorological conditions is reported in Figure 1.

### 2.2. Canopy Architecture

**Figure 2:**
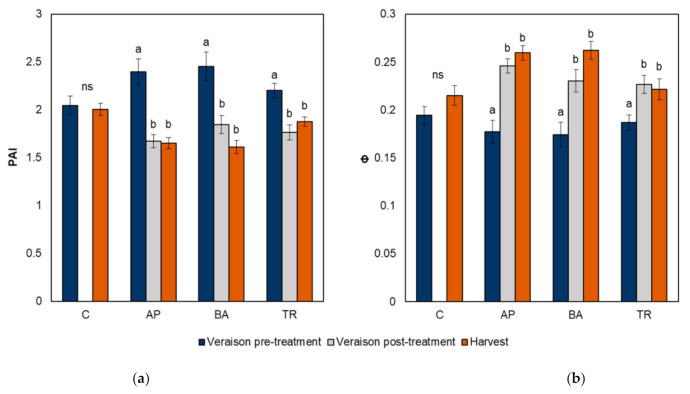
Effect of leaf removal treatments on the plant area index, PAI (**a**) and canopy porosity, Φ (**b**) measured before and after treatment and at harvest using the VitiCanopy App. AP = apical leaf removal, BA = basal leaf removal, TR = trimming, and C = control. Means were separated by ANOVA (*p* ≤ 0.05), and different letters indicate significant differences within each treatment group. ns = not significant.

By imaging the vines with the VitiCanopy App [24] before and after the treatments were applied it was possible to make a pre and post-treatment comparison of the immediate effect of the treatments on vine canopy architecture. A significantly lower plant area index (PAI), and a higher porosity (Φ) were observed after the application of all three defoliation techniques. It was found that shoot trimming the eastern side of the canopy to five or six nodes (TR) reduced the PAI on average by approximately 20%, while traditional basal leaf removal applied around the fruit zone on the eastern side of the canopy (BA) and leaf removal applied to the top two-thirds of the shoots apical to the bunches (AP) reduced the PAI on average by approximately 25% and 30%, respectively (Figure 2). In regard to canopy porosity, Φ increased on average by approximately 21% after treatment in TR vines, 32% in BA vines, and 39% in AP vines. The PAI and Φ of all treatments were different from control vines immediately after treatment application.

Vines were re-imaged on the date of harvest. No difference was observed between the PAI or Φ values measured at veraison (post-treatment) and at harvest for each treatment (Figure 2). The PAIs of apically defoliated and basally defoliated vines were lower than control and shoot trimmed vines at harvest (*p* < 0.0001).

### 2.3. Vine Performance

Yield and yield components did not vary between treatments (Table 1). Total leaf area per plant at harvest was lower for the apical and basal leaf removal treatments than shoot trimmed and control vines. The LA/Y ratio was lower in apically defoliated vines than shoot trimmed and control vines.

### 2.4. Grape Chemistry and Sensory Attributes

**Figure 3:**
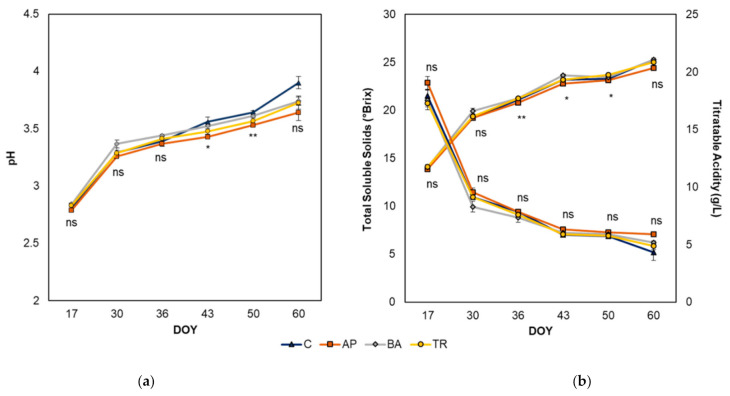
Effect of leaf removal treatments on grape pH (**a**), titratable acidity, and total soluble solids (**b**). AP = apical leaf removal, BA = basal leaf removal, TR = trimming, and C = control. *, ** indicate significant differences at *p* ≤ 0.05 and 0.01, respectively. Means were assessed using ANOVA. ns = not significant. DOY, day of year.

On the date of harvest, no difference in total soluble solids (TSS) was observed across treatments, with values ranging from 24.4 to 25.3 °Brix (Figure 3). No difference in titratable acidity (TA) was observed across treatments at harvest or on any other sampling date. Although no difference was observed in pH at harvest, a higher pH was observed in apically defoliated and shoot trimmed vines compared to control vines on day of year (DOY) 43 and DOY 50.

**Figure 4:**
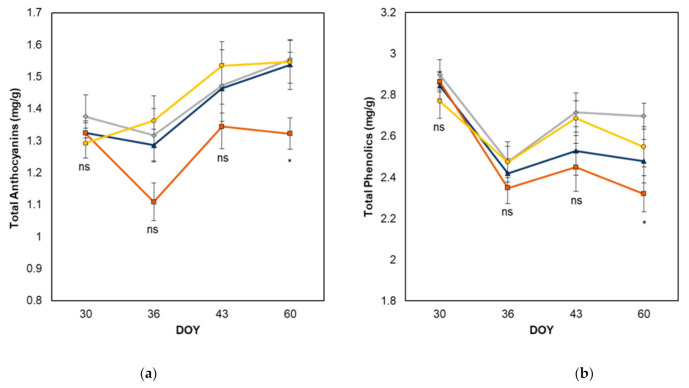

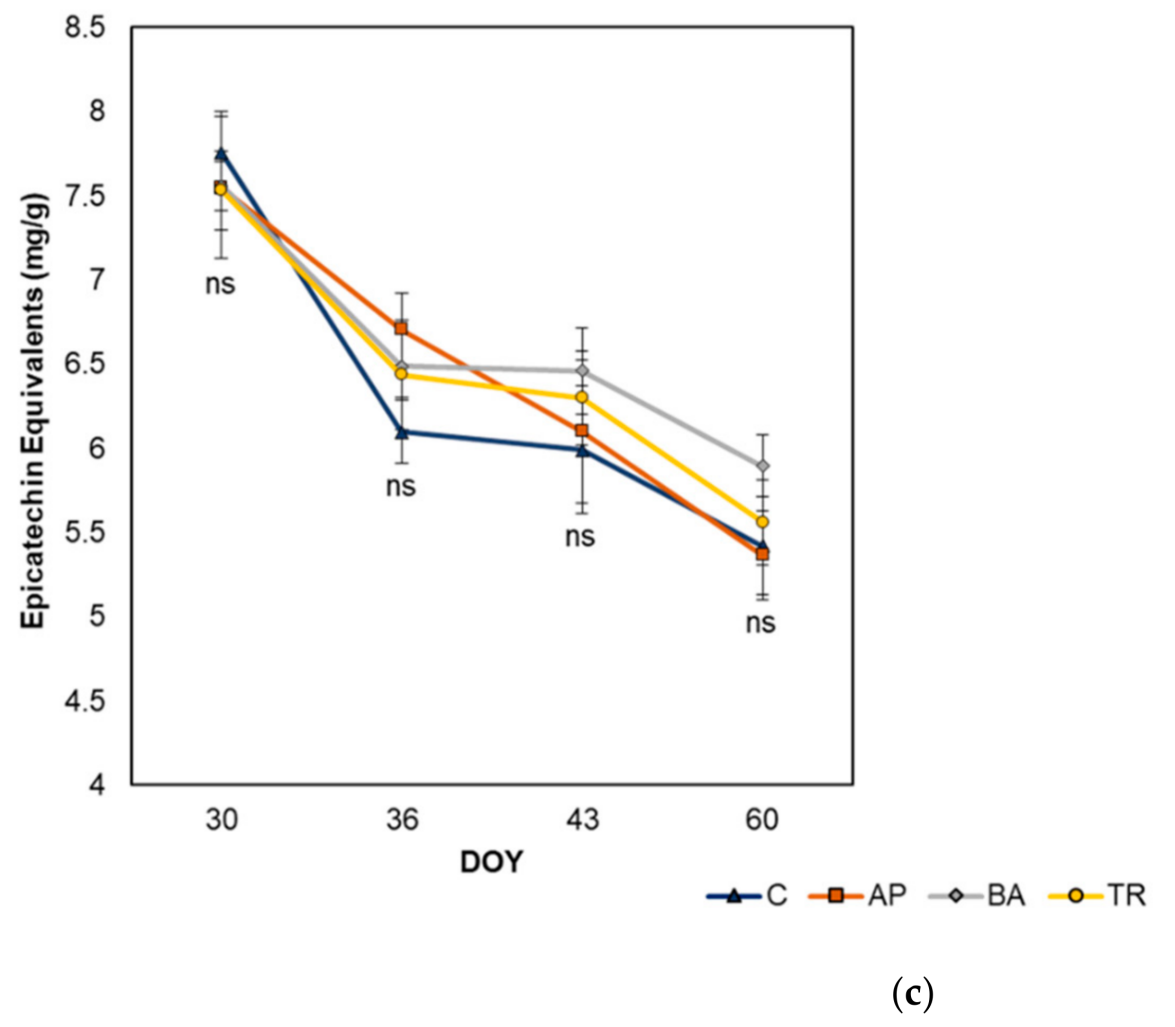
Effect of leaf removal treatments on grape total anthocyanins (**a**), total phenolics (**b**), and total tannins (**c**) (expressed as epicatechin equivalents). AP = apical leaf removal, BA = basal leaf removal, TR = trimming, C = control. * indicates significant differences at *p* ≤ 0.05. Means were assessed using ANOVA. ns = not significant. DOY, day of year.

The concentration of grape anthocyanins was lower in apically defoliated vines compared to control vines and other treatments at harvest (Figure 4). No difference was observed in anthocyanin levels across other sampling dates. At harvest, the concentration of grape phenolics was lower in apically defoliated vines compared to basally defoliated vines, although no difference was observed between the control and other treatments at harvest or on any other sampling date. The concentration of grape tannins did not vary among treatments at harvest or on any other sampling date.

**Figure 5:**
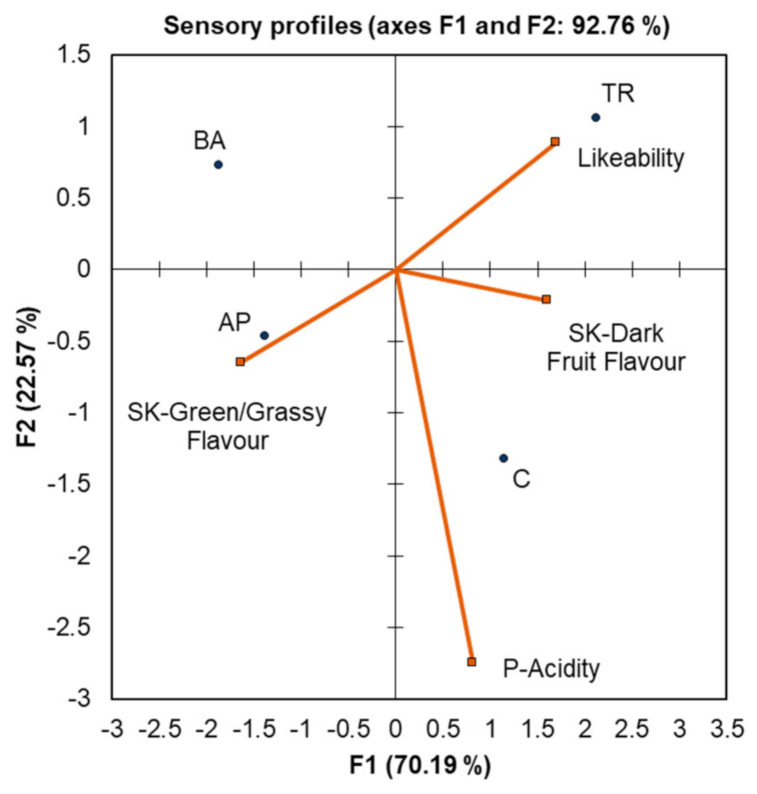
Principal component analysis of the attributes found significantly different at *p* ≤ 0.1 by panel assessment in the berries from different leaf removal treatments. AP = apical leaf removal, BA = basal leaf removal, TR = trimming, C = control. P-Acidity = acidity of the pulp, SK-Dark Fruit Flavour = dark fruit flavour of the skins, and SK-Green/Grassy Flavour = green/grassy flavour of the skins.

Berry sensory assessment (BSA) was performed by a trained panel of 11 assessors. The first two principal components (PCs) in the PCA in Figure 5 explain 92.76% of the variation in the dataset. PC 1 separates shoot trimmed and control vines from basally and apically defoliated vines. The basal and apical leaf removals are associated with green grassy flavours in the skin while shoot trimmed was the most liked. Both control and shoot trimmed vines were described as having higher dark fruit flavour in the skin. The separation of the treatments along PC 2 is mostly due to the acidity of the pulp which was perceived as being higher in the control.

## 3. Discussion

The impact of the different defoliation methods on canopy architecture was quantified using the plant area index (PAI) and canopy porosity (Φ), where the PAI describes the total one-sided area of leaf tissue per unit ground surface area [25] and Φ refers to the light penetration through the canopy [24]. A significantly lower PAI and higher porosity (Φ) were observed after the application of all three treatments, with the greatest reduction in the PAI (30%) taking place on apically defoliated vines. This is contrary to the results of Zhang et al. [15], who in their study on Shiraz observed a significant reduction in the PAI and increase in Φ on vines which were basally defoliated but not those which were apically defoliated. This is likely explained, however, by the fact that their study was conducted on a vertically shoot positioned (VSP) system in contrast to this study which was conducted on a sprawl system and as such the shoots being defoliated in this trial were not constrained vertically to the top portion of the canopy and therefore the removal of apical leaves had a much greater effect on canopy architecture and shading. No significant difference was observed between PAI or Φ values measured post-treatment at veraison and at harvest for all treatments. This indicates that there was no compensation in leaf area between when the treatments were performed and harvest. Interestingly, total leaf area per plant at harvest was lower than the control only for the apically and basally defoliated vines and not shoot trimmed vines. While this could be taken to suggest that there was some compensatory growth of lateral shoots stimulated by the trimming, the fact that the same vines imaged both immediately post-treatment application and at harvest showed no change in the PAI indicates that there was minimal to no leaf surface area recovered during this time period. This agrees with the results of another study which found lateral growth to be poor after late season trimming of Aglianico vines at ~12 °Brix [26], and is likely due in part to the fact that at this stage of development grape sugar accumulation is rapidly increasing and the berries are a stronger sink for carbon than shoots [27].

While it has been reported that trimming may cause a reduction in yield when applied around berry set [28,29], at veraison [30], or post-veraison [31], in this study yield and yield components did not vary significantly between treatments. The LA/Y ratio was, however, found to be lower in apically defoliated vines (1.5 m^2^/kg), than shoot trimmed (2.2 m^2^/kg) and control (2.6 m^2^/kg) vines at harvest. It has been suggested that grape sugar accumulation rate depends on this ratio and that values ranging between 0.8 and 1.2 m^2^/kg are ideal for producing good fruit and wine quality on a single-canopy training system [14]. This “optimal” value of the ratio may, however, not be universal, with climate, variety, and location being important factors of consideration as the results of some studies have indicated [1,22,26]. Despite the reduction in LA/Y observed in apically defoliated vines in this trial, on the date of harvest no significant difference in grape TSS was observed between treatments. Although it is well known that the accumulation of sugar is dependent on the active leaf area available during the period between veraison and harvest, it appears that the source limitation induced by the three defoliation methods in this trial did not significantly affect the accumulation of grape TSS. This is contrary to the results of numerous other studies who reported a reduction in the rate of sugar accumulation after apical leaf removal [1,15,20,29,32] and shoot trimming [28,29,30,31,33,34,35,36,37,38]. This may be related to the fact that with the 3 m inter-row spacing of the vineyard of study, light was not a limiting factor. It may also be related to the fact that the vines of this trial had a higher starting leaf area due to the 2 m intra-row spacing of the vineyard, and thus the reduction observed in the PAI after the treatments did not have as great of an impact on carbohydrate source limitation.

No difference in TA was observed between treatments on any sampling date. Although no difference was observed in pH at harvest, a higher pH was observed in apically defoliated and shoot trimmed vines compared to control vines on DOY 43 and DOY 50. This somewhat agrees with the results of Zhang et al. [15] who reported that apical defoliation near veraison resulted in an increase in pH and decrease in TA on Shiraz. In contrast however, several studies have reported a severe shoot trimming between fruit set and veraison [28,30,33] and post-veraison [31] to cause a reduction in pH, while others have shown pH to remain unaffected in apically defoliated [29] and shoot trimmed [29,34] vines when treatments were applied between fruit set and veraison.

The concentration of grape anthocyanins was lower in apically defoliated vines compared to control vines at harvest, with no difference observed in anthocyanin levels between treatments across other sampling dates. This somewhat correlates with the concentration of grape phenolics which was found to be lower in apically defoliated vines compared to basally defoliated vines at harvest, although no difference was observed between the phenolics of control vines and other treatments at harvest or on any other sampling date. Similar results were obtained by Zhang et al. [15], who reported in their two-vintage study that apical defoliation resulted in a reduction in grape anthocyanins and wine colour profile compared to control vines in one growing season, but not the other. The reductions observed in the anthocyanin and phenolic levels may be related to the fact that the AP vines showed the greatest decrease in the PAI (30%) and increase in Φ (39%) after treatment. It is well known that the synergistic effects of both light and temperature are crucial for anthocyanin synthesis and that elevated temperatures and severe UV exposure resulting from canopy opening can result in the inhibition of the biosynthesis of anthocyanins and other important phenolic compounds [18]. One would expect however, given the proximity of the leaves being removed to the fruit zone, that the basally defoliated vines would show a greater change in bunch microclimate compared to those that were apically defoliated, despite the changes to the PAI and Φ being slightly more minimal (a decrease of 25% and increase of 32%, respectively). Total grape tannins were measured spectrophotometrically using the methyl cellulose precipitable (MCP) assay proposed by Mercurio et al. [39], which has been utilised successfully in previous experiments to determine differences in tannin levels between various canopy management treatments [22]. The concentration of grape tannins did not vary significantly among treatments at harvest or on any other sampling date. This agrees with the results of Filippetti et al. [34] who reported a severe trimming at 12 °Brix reduced TSS at harvest by one °Brix without affecting pH, TA or the concentration of anthocyanins and tannins in Sangiovese.

The results of the BSA are somewhat inconclusive, although it is clear that green/grassy flavours are more pronounced in the apically defoliated and basally defoliated treatments and are separated from the dark fruit flavour which is more pronounced in the control and shoot trimmed vines. This is surprising, as one might expect the less developed green/grassy flavours to be less pronounced in the leaf removal methods which saw a greater reduction in the PAI and provided greater bunch exposure. It is also particularly interesting when one considers that the apically defoliated vines displayed less anthocyanin development than the other treatments, another characteristic normally typical of less mature fruit. It is possible that these treatments could reduce the development of riper fruit flavours as their sampled fruit also displayed less dark fruit flavour in the skins. These differences observed between treatments may be related to the similarity of the basal and apical leaf removal treatments wherein only leaves were removed and not actively growing shoot tips as was the case with the shoot trimmed vines. The pulp of the grapes from control vines was perceived as being more acidic than that of the three defoliation treatments, which does not correlate with the values measured for pH and TA at harvest where no difference was found between treatments. These results highlight the importance of assessing not only berry chemistry, but also sensory attributes, currently uncommon in studies of this nature. Grapes from shoot trimmed vines were found to have the greatest overall likeability. This may be more related to the removal of the shoot tips (a major sink for nutrients and energy) than to a reduction in active leaf area, as the change in the PAI observed after treatment (20%) was lower than that of the other two leaf removal treatments. The PAI did not change between trimming and harvest, indicating that there was not a substantial compensatory growth of lateral shoots induced by the treatment. Further research should be focused on a shoot trimming applied earlier in the growing season, allowing enough time for adequate growth of lateral shoots to provide additional competition to the developing berries as a sink for carbon.

While various other studies have shown the potential to achieve slower grape sugar accumulation without affecting the concentration of anthocyanins, phenolics, and tannins, the results of this study do not indicate a decrease in the rate of grape sugar accumulation as a result of the investigated defoliation techniques. This is despite the fact that the treatments had a significant impact on the PAI and Φ, and may have more to do with the site details (non-light-limiting environment) than with differences in treatments. Other results including the effect of the treatments on acidity, anthocyanins, phenolics, and tannins are somewhat inconclusive. In regard to the economy of the use of these treatments by growers, the results of this study do not support the use of these methods for the purpose of delaying fruit ripening in a hot Australian climate, particularly as another recent study yielded similar results with different cultivars [22]. In order to obtain more conclusive results, further research conducted during consecutive growing seasons is needed. Applying the shoot trimming treatment at the same intensity earlier in the growing season would allow more time for adequate growth of lateral shoots.

## 4. Materials and Methods

### 4.1. Experimental Site

The trial was carried out in the 2017–2018 growing season in an irrigated commercial vineyard located in McLaren Vale, South Australia (35°11′39.7″ S; 138°31′10.4″ E). The vineyard was a 12-year-old planting of Cabernet Sauvignon (clone CW44), grafted onto 110 Richter rootstock, and planted at 3 m × 2 m inter-row and intra-row with a north–south row orientation. Vines were trained to a spur-pruned cordon trellis in a sprawl system with a bud load of 40 nodes per vine. The total amount of irrigation discharged was ~0.8 ML/ha during the growing season, the same rate applied the previous 3 years. The climatic conditions for the site were sourced from the nearest Australian Bureau of Meteorology (http://www.bom.gov.au/, accessed on 10 June 2018) weather station located in Noarlunga (station number 23885).

### 4.2. Experimental Design

The trial was set up in three rows in a randomised block design, with each row as a block. Treatments were randomly allocated along each row in two replicates of six vines per treatment. A total of 36 vines per treatment were used. Each of these rows was spaced with a buffer row between them. Treatments included (i) control (C), where no canopy intervention was performed; (ii) leaf plucking in the bunch zone (BA), where five to seven leaves were removed from the basal portion of each shoot on the eastern side of the canopy, as is standard industry practice; (iii) leaf plucking on the top two-thirds (AP), where leaves were removed on each shoot apical to the bunches, leaving 3–4 leaves immediately above the bunches and 3–4 leaves at the apex of the shoot; and (iv) shoot trimming (TR), where each shoot on the eastern side of the canopy was cut down to approximately 5–6 leaves, considered a severe trimming. Treatments were performed at veraison (~14 °Brix), on DOY 17.

### 4.3. Canopy Architecture, Yield Components and Grape Composition

Before treatments were carried out images were taken with a smartphone for the purpose of measuring canopy architecture using the VitiCanopy App [24]. One image was taken on each side of the middle vine of each panel from approximately 80 cm below the vine cordon. These same vines were also re-imaged just after the treatments were applied to allow for a pre and post-treatment comparison. On the date of harvest (DOY 60), the vines were imaged a final time.

Random samples of 120 berries per treatment and per block were collected on a total of six dates between DOY 17 and DOY 60 and returned to the lab, where a random subsample of 50 berries was collected to measure berry weight and then stored at −20 °C for 8 weeks to be used in the analysis of phenolic and tannin content. Total grape tannins were measured spectrophotometrically using the methyl cellulose precipitable (MCP) assay proposed by Mercurio et al. [39]. Total anthocyanins and phenolics were determined according to Iland et al. [40]. The remaining berries were crushed by hand in plastic bags with the juice then being collected in 50 mL tubes and centrifuged at 5000 rpm for 5 min (Hettich Universal, Tuttlingen, Germany) before total soluble solids (TSS), pH and titratable acidity (TA) were measured according to Iland et al. [40], using an automatic titrator (G20S Compact Titrator, Mettler Toledo, Thebarton, Australia) and a digital refractometer (BRX-242 Erma Inc. Tokyo, Japan). On DOY 60, all of the treatments were hand harvested and the number of bunches and yield per vine were recorded. Cordon length was also measured so that yield and its components could be determined on a per metre basis. From yield and bunch number, the average bunch weight was calculated.

### 4.4. Berry Sensory Analysis

At harvest, 120 berries of similar size were cut from a sample of 10 bunches per treatment and per block with scissors, leaving the pedicel on the berries in order to limit oxidation and deterioration. These berries were then stored at −20 °C for 12 weeks for the purpose of berry sensory assessment (BSA) following the procedure described in Lohitnavy et al. [41] and Olarte Mantilla et al. [42]. BSA was carried out at the sensory facility of the Plant Research Centre at the University of Adelaide Waite Campus with the approval of the University of Adelaide ethics committee (H-2018-2008). A group of 11 assessors with previous BSA experience were trained over a one-hour session where the attributes to score during the formal assessment were decided. During the training sessions, the assessors were asked to evaluate berry samples with the aim of reaching an agreement on the number and type of attributes to score during the formal sessions. The list of attributes was then created on a 0–15 line scale with two anchors to be used in the assessment. The descriptors were divided into pulp, skin and seeds characteristics (Table 2).

During the formal assessment sessions, each of the panelists were presented with 24 three-berry samples in a randomised presentation order for each assessor in two sessions of 12 samples each. Each sample was evaluated twice by each assessor. A custom-designed app was used for sample assessment purposes. Attributes were presented in the order shown in Table 2 and each attribute was associated to a line scale with a cursor that the assessor could slide by tapping on the screen of a tablet or smartphone. The application collated the data for each session in an excel file with the sample name in the rows and the assessor name and all of the attributes in the columns. After the completion of the assessment an excel file containing all of the information collected was sent to a nominated email account and downloaded for analysis.

### 4.5. Statistical Analysis

ANOVA and principal component analysis (PCA) were performed using XLSTAT Version 2018.3 (Addinsoft SARL, Paris, France). Sensory data were analysed as a two-way mixed-models ANOVA with random assessors.

## Figures and Tables

**Table 1:** Effect of leaf removal treatments on yield and yield components (mean ± std).

Treatment	Yield (kg/m)	Bunch No. (no./m)	Bunch Weight (g)	Berry Weight (g)	Total Leaf Area (m^2^/m)	LA/Y (m^2^/kg)
Control	2.4 ± 0.7	32 ± 6.9	72.8 ± 10.1	0.78 ± 0.05	5.8 ± 1.1	2.6 ± 0.8
Apical leaf removal	3.3 ± 1.2	40 ± 5.5	80.5 ± 25.2	0.89 ± 0.06	4.6 ± 1.0	1.5 ± 0.4
Basal leaf removal	2.5 ± 1.2	32 ± 12.1	75.2 ± 10.9	0.88 ± 0.12	4.4 ± 1.1	2.1 ± 1.0
Shoot trimming	2.6 ± 0.6	32 ± 7.5	81.1 ± 12.3	0.83 ± 0.03	5.3 ± 0.8	2.2 ± 0.7
Significance	ns	ns	ns	ns	<0.0001	0.018

Means were separated by ANOVA (*p* ≤ 0.05). ns = not significant. LA/Y, leaf area/yield.

**Table 2:** List of descriptors/attributes assessed by the panelists during the BSA with their scale left and right anchor. The attributes were divided in four categories: pulp characteristics, skin characteristics, seed characteristics and overall likeability.

Attribute	Left Anchor	Right Anchor
Pulp Characteristics		
Sweetness	Low	High
Acidity	Low	High
Dark Fruit Flavour	Low	High
Dried Fruit Flavour	Low	High
Flavour Intensity	Low	High
Skin Characteristics		
Disintegration	Low	High
Acidity	Low	High
Dark Fruit Flavour	Low	High
Green/Grassy Flavour	Low	High
Astringency	Low	High
Seed Characteristics		
Colour	Green	Dark Brown
Flavour	Herbaceous	Toasted
Bitterness	Low	High
Astringency	Low	High
Overall Likeability	Low	High

## Data Availability

The data presented in this study are available on request from the corresponding author. All tables and figures presented are original.
